# The 1-Hour Plasma Glucose: Common Link Across the Glycemic Spectrum

**DOI:** 10.3389/fendo.2021.752329

**Published:** 2021-09-07

**Authors:** Michael Bergman

**Affiliations:** Division of Endocrinology, Diabetes and Metabolism, VA New York Harbor Healthcare System, NYU Grossman School of Medicine, New York, NY, United States

**Keywords:** prediabetes, type 1 diabetes, type 2 diabetes, gestational diabetes, oral glucose tolerance test (OGTT)

## Abstract

Evidence from populations at risk for type 1 diabetes, type 2 diabetes or gestational diabetes substantiates the 1-hour plasma glucose as a sensitive alternative marker for identifying high-risk individuals when ß-cell function is relatively more functional. An elevated 1-hour plasma glucose could therefore diagnose dysglycemia and risk for complications across the glycemic spectrum. Reducing the 2-hour oral glucose tolerance test to 1-hour would reduce the burden on patients, likely reduce costs, and enhance its accessibility in practice.

## Introduction

The heterogeneity of diabetes has been the subject of considerable discussion given the complexity of classifying diabetes further mitigated by genetics and environmental factors (1, 2). While type 1 diabetes is generally diagnosed based on the presence of antibodies and type 2 diabetes by obesity and insulin resistance, it has become increasingly evident that younger individuals with type 1 diabetes may be insulin resistant and overweight or obese adolescents can have type 2 diabetes and the metabolic syndrome (1). Alternatively, adults with type 2 diabetes may have antibodies and manifest type 1 diabetes more insidiously than in childhood (1, 2). Ahlqvist et al. highlighted the complexity of classifying patients using clustering methodology with six clinically accessible parameters and found five clusters of patients demonstrating different clinical trajectories concerning initiation of therapy and development of complications (3, 4). The criteria included glutamate decarboxylase antibodies (GAD) antibodies to identify autoimmune diabetes, homeostatic model assessment 2 (HOMA2)-B assessing β-cell function with C-peptide measurements and HOMA2-IR to assess insulin sensitivity. Hence, β-cell function in the pathogenesis of diabetes is critical to both type 1 and type 2 diabetes. The Saku study involving 3,059 Japanese participants aged 30-69 without diabetes at baseline found that after 4 years, the population-attributable fraction of type 2 diabetes was 50.6% in those with isolated β-cell dysfunction and 14.2% in those with isolated insulin resistance thereby confirming the importance of β-cell dysfunction in the pathogenesis of type 2 diabetes (5).

**Figure 1:**
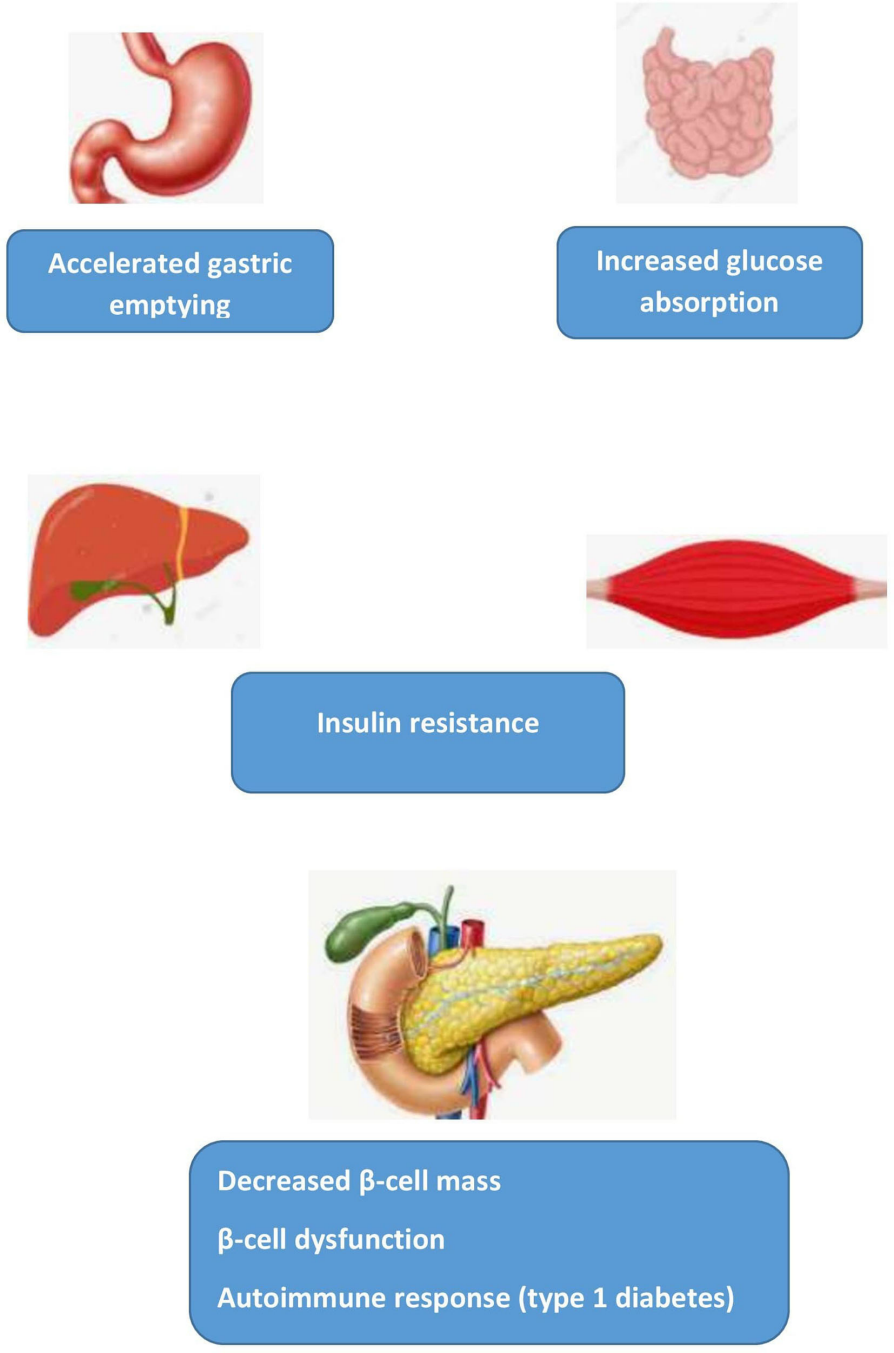
Elevated 1-hour plasma glucose: “common soil” pathophysiological mechanisms. Adapted from Fiorentino et al. (6).

These considerations have led to the formulation of a “β-cell centric classification” system grounded in the realization that the “common soil” for the diagnosis of diabetes relates to pancreatic β-cell dysfunction and decreased β-cell mass interacting with diverse contributing factors including genetic predisposition, insulin resistance, environment, microbiome, immune dysregulation and inflammation (**Figure 1**) (7). Focusing on the β-cell as the root cause underlines the necessity for early preventive initiatives during the lengthy prediabetes stage when the β-cell is more apt to respond to intervention and consequently make reversibility more likely. As remission becomes increasingly improbable with duration of disease, it is critical to reduce progression to type 2 diabetes or intervene vigorously shortly after diagnosing type 2 diabetes. Duration of diabetes was the major determining factor in achieving remission with weight loss in the primary care setting in the Diabetes Remission Clinical Trial (DiRECT) (8, 9). Furthermore, in the UKPDS, which recruited individuals with newly diagnosed type 2 diabetes, early detection by screening of diabetes and maintenance of intensive glucose control from the time of diagnosis rather than delaying by 10 years was essential to maximize reduction of the long-term risk of all-cause mortality and myocardial infarction (10). As many patients are not treated intensively either at disease onset or later in its course, diabetes prevention therefore becomes increasingly essential to avoid developing diabetes-related complications including end-stage renal disease despite the availability of effective pharmacotherapy that can reduce the incidence of the latter (11).

Therefore, given the essentiality of preserving β-cell function to thwart progression to diabetes regardless of type, it becomes increasingly important to identify early biomarkers in those at high-risk. This is particularly important given the recent acknowledgement that glycemic control has declined since 2010 (12) with a resurgence of diabetes-related complications (13) occurring in young and middle-aged adults. Furthermore, without measuring the 2-hour plasma glucose, HbA1c and fasting plasma glucose alone or combined underestimate the prevalence of undiagnosed diabetes (14). This observation emphasizes the importance of the oral glucose tolerance test (OGTT) in surveillance of diabetes (14). In this context, the 1-hour plasma glucose during an OGTT has been shown to be a novel and more sensitive biomarker for predicting progression to both type 1 and type 2 diabetes (15–17) in individuals with normal glucose tolerance and more sensitive than the 2-hour plasma glucose for diagnosing type 2 diabetes (18). Finally, the 1-hour plasma glucose during pregnancy may identify high-risk women with increased adverse maternal and perinatal outcomes and is an independent predictor of type 2 diabetes and future cardiovascular disease (19).

## 1-Hour Plasma Glucose – Type 1 Diabetes

Islet autoantibody–positive individuals may be eligible to participate in diabetes prevention trials so it is vital to identify markers that can predict progression from autoantibody positivity to type 1 diabetes (15). The 2-hour oral glucose tolerance test (OGTT) has been useful in predicting risk for type 1 diabetes (20, 21) in the TrialNet Pathway to Prevention (TNPTP) study and the Diabetes Prevention Trial-Type 1 (DPT-1). However, as the 2-hour OGTT is burdensome with regard to time and cost, the 1-hour OGTT can predict risk of progression to type 1 diabetes with greater convenience (15).

The Diabetes Prevention Trial Risk Score (DPTRS), based on data obtained within 2 hours of a glucose challenge, was modified to derive a 1-hour OGTT risk score (DPTRS60) comprising fasting C-peptide, 1-hour glucose and C-peptide, age, and body mass index. The DPTRS60 was more accurate than the DPTRS, the standard measurement, for predicting type 1 diabetes than the 2-hour glucose in a study validated in two independent cohorts of autoantibody positive individuals at risk for type 1 diabetes (15). A 1-hour glucose threshold of 180 mg/dL (10.0 mmol/L) or greater had a similar negative predictive value and specificity to a 2-hour glucose of 140 mg/dL (7.8 mmol/L) or greater and could be used for surveillance of type 1 diabetes (15). The authors concluded that the 2-hour OGTT be shortened to 1 hour and be reliably implemented for evaluating individuals at risk for type 1 diabetes participating in research.

Index60 (22) was developed as a metabolic diagnostic indicator of type 1 diabetes and does not include age and BMI as the DPTRS. Index60, derived from a 1-hour OGTT with two blood samples, is a composite of fasting C-peptide, 60-minute C-peptide and glucose measurements. It identifies individuals with characteristics of type 1 diabetes at high- risk for stage 3 (clinical diabetes) among those autoantibody positive (Ab+) (23). Therefore, Index60 can be utilized for predicting type 1 diabetes as it correlates with the DPTRS (24). In DPT-1, the Index60 threshold ≥2.00 occurred approximately one year before a diagnosis made based on standard OGTT criteria. In TNPTP as well, an Index60 value ≥2.00 identified more individuals with characteristics typical of type 1 diabetes than glucose criteria (25). These findings suggest that the Index60 threshold ≥2.00 could diagnose type 1 diabetes earlier (24, 25). Individuals reaching the Index60 threshold ≥2.00 with a 2-hour glucose value < 200 mg/dL (11.1 mmol/L) appear to approach the stage of clinical type 1 diabetes (stage 3) (23). The Index60 with a lower threshold value ≥ 1.00 may serve as an earlier pre-diagnostic marker (i.e., before the occurrence of type 1 diabetes). Incident dysglycemia in the absence of Index60 ≥1.00 was found suboptimal in the TNTPT as a prediagnostic end point for type 1 diabetes. Measures that include both glucose and C-peptide levels, such as Index60 ≥1.00, therefore are better prediagnostic end points (26). These observations provide evidence that diagnostic criteria for type 1 diabetes should include Index60 or a similar index (25).

## 1-Hour Plasma Glucose – Type 2 Diabetes

As early detection of dysglycemia in high-risk individuals may avert progression to diabetes, development of microvascular complications and mortality, identifying the earliest moment for intervention is critical although challenging. As fasting and postprandial glucose deteriorate insidiously in a gradual, continuous process from normal glucose tolerance to prediabetes and type 2 diabetes, implementing lifestyle intervention when the β-cell is substantially more functional before achieving the prediabetes stage could be even more effective in preventing progression to diabetes. Hence, as seen for type 1 diabetes, earlier detection of those at risk with more sensitive biomarkers is essential (27, 28).

The 1-hour post-load plasma glucose during the OGTT in those at high-risk for type 2 diabetes has been widely reviewed (6, 17, 29). The 1-hour post-load plasma glucose is characterized by (1) optimal specificity and sensitivity, (2) the ability to diagnose high-risk individuals with normal glucose tolerance early in the trajectory to diabetes before β-cell functionality is substantially impaired, and (3) to predict progression to type 2 diabetes, complications and mortality. The prevalence of an elevated 1-hour plasma glucose in those with normal glucose tolerance ranges from 11–16% in population-based observational studies to ~25-42% in high-risk cohorts with at least one cardiovascular risk factor. The prevalence increases as glucose tolerance deteriorates with > 50% of individuals with combined impaired fasting glucose and impaired glucose tolerance and > 90% of newly diagnosed type 2 diabetes having elevated 1-hour plasma glucose levels (6).

Factors that account for the increased proximal intestinal absorption of glucose observed during the OGTT explaining the elevated 1-hour plasma glucose have been considered in the literature (30). Gastric emptying accounts for approximately one-third of the variance in peak plasma glucose levels during a 75-g in healthy individuals (31). Furthermore, plasma glucose levels appear to be inversely related to gastric emptying at 120 minutes. Alterations in gastric emptying may therefore explain variability in glucose concentrations during the OGTT (31). For example, rapid gastric emptying was observed at 60 minutes during an OGTT in individuals having impaired glucose tolerance (IGT) and type 2 diabetes but not in those with normal glucose tolerance (30). An inverse relationship between glucose and gastric emptying occurred at 120 minutes in those with normal glucose tolerance but not in those with IGT or type 2 diabetes (30).

In a recent study, gastric emptying was delayed in ageing individuals without diabetes but was more rapid in patients with type 2 diabetes of short duration regardless of control (32). These findings confirm observations from an earlier study of individuals with well-controlled type 2 diabetes of short duration (33). Finally, these physiologic findings have clinical consequences as rapid gastric emptying of a liquid glucose meal determined by scintigraphy in recently diagnosed asymptomatic individuals with type 2 diabetes could explain the poor metabolic control frequently observed in this population (34).

Another mechanism for the increased proximal intestinal absorption of glucose relates to the sodium/glucose cotransporter 1 (SGLT-1). An elevated 1-hour plasma glucose during the early phase of the OGTT may be attributable to increased expression of SGLT-1 in the proximal intestine. Fiorentino et al. have shown that duodenal SGLT-1 expression was significantly higher and correlated with an elevated 1-hour plasma glucose (6, 35).

The 1-hour plasma glucose level is a better alternative to HbA1c [5.7–6.4% (37–47 mmol/mol)] or traditional glucose criteria (fasting, 2-hour plasma glucose) for identifying high-risk individuals when ß-cell function is substantially more intact than in prediabetes (27). Pathophysiological abnormalities associated with a 1-hour plasma glucose ≥ 155 mg/dL (8.6 mmol/L) include impaired insulin sensitivity and β-cell function. Tura et al. recently demonstrated that individuals with an isolated elevation in the 1-hour plasma glucose showed differences in insulin sensitivity, insulin clearance, glucose sensitivity, glucose potentiation factor and β-cell function compared to other groups with isolated defects [isolated impaired fasting glucose and isolated HbA1c). An isolated elevation in the 1-hour plasma glucose could therefore identify a prediabetes phenotype with worse β-cell function (36)].

Considerable epidemiologic evidence derived from different populations substantiates that the elevated 1-hour plasma glucose level has greater sensitivity for identifying high-risk individuals with normal glucose tolerance. The 1-hour plasma glucose has been associated with cardiovascular risk factors in the CATAnzaro Metabolic RIsk factors (CATAMERI) study including higher BMI, abdominal obesity, atherogenic lipid pattern, uric acid, apolipoprotein levels, and elevated levels of inflammatory and coagulation factors. This study has also provided mechanistic insights into the association of the 1-hour plasma glucose with subclinical target organ damage such as carotid atherosclerosis, cardiac insulin resistance, fatty liver, and impaired kidney function. These observations may explain the greater risk for hepatic steatosis, micro- and macrovascular complications, cardiovascular disease (CVD) and mortality in those with normal glucose tolerance and a 1-hour plasma glucose ≥155 mg/dL (8.6 mmol/L) (6, 16, 17).

The considerable evidence base led to the formulation of a published petition requesting that scientific organizations consider the 1-hour plasma glucose for diagnosing prediabetes (17). More recently, a meta-analysis of the 1-hour plasma glucose for diagnosing type 2 diabetes identified the threshold of ≥ 209 mg/dL (11.6 mmol/L) with good sensitivity and specificity for detecting type 2 diabetes. Implementation of a diabetes-specific risk calculator for prescreening high-risk individuals could decrease the proportion of false positive cases (18). The 1-hour OGTT could therefore be employed to both detect individuals at high-risk for type 2 diabetes and diagnosing type 2 diabetes.

Finally, an elevated 1-hour plasma glucose (≥155 mg/dL [8.6 mmol/L]) identified dysglycemia and glycemic variability (GV) measured by continuous glucose monitoring (CGM) in normal glucose tolerant subjects (HbA1c <5.7% [39 mmol/mol]) at high-risk for diabetes (37). Since GV has been associated with increased risk for diabetes and complications, early detection with the 1-hour plasma glucose or 1-hour CGM interstitial glucose during an OGTT in high-risk individuals with normal glucose tolerance is therefore essential.

## 1-Hour Plasma Glucose – Gestational Diabetes Mellitus

Identifying high-risk women with gestational diabetes mellitus (GDM) is important as it is associated with a seven-fold greater risk of developing type 2 diabetes and a two-fold greater risk of developing CVD (38, 39). The Hyperglycemia and Adverse Pregnancy Outcome (HAPO) study showed a highly significant association between maternal fasting and post-load glucose values and increased birth weight as well as higher cord-blood serum C-peptide levels (32). Moreover, premature delivery, intensive neonatal care, and hyperbilirubinemia related significantly to 1- and 2-hour plasma glucose levels but not the fasting plasma glucose. The 1-hour plasma glucose was a significant independent predictor of clinical neonatal hypoglycemia (39) and a threshold of 160 mg/dL (8.9 mmol/L) could identify women with increased risk of fetal hyperinsulinemia and adverse clinical outcomes. Children whose mothers had a 1-hour value between 160 (8.9 mmol/L) and 179 mg/dL (9.9 mmol/L) had significantly higher cord blood insulin values (40). The HAPO study documented a significant association between 1-hour values and abnormal neonatal anthropometric features (cranial/thoracic circumference, ponderal index, macrosomia) and provided evidence of a threshold relation between 75-g glucose load results and clinical outcome (41). The 75-gram 1-hour oral glucose load could serve as a single test for the diagnosis of GDM adopting a threshold value of 150 mg/dL (8.3 mmol/L) at 16–20 weeks and 160 mg/dL (8.9 mmol/L) at 26–30 weeks (42).

An abnormal 1-hour value is associated with increased glycemia, insulin resistance, and β-cell dysfunction both in pregnancy and at 3 months postpartum. Furthermore, its independent association with β -cell dysfunction suggests that the 1-hour value may predict increased future risk of type 2 diabetes and thereby identify a high-risk population necessitating postpartum surveillance (35). In pregnant women *not* meeting the criteria for GDM, the 1-hour plasma glucose was an independent predictor of postpartum impaired insulin sensitivity and β-cell dysfunction (43). Hyperglycemia at 1-hour during pregnancy may identify women at high-risk of future CVD related to its association with an unfavorable CV risk profile, inflammation, arterial stiffness and endothelial dysfunction. In summary, an elevated 1-hour plasma glucose in pregnancy may be valuable for identifying high-risk women who may benefit from interventions targeting preventing progression to type 2 diabetes and CVD.

There is also novel evidence for a continuous relationship of increasing maternal glucose levels below the diagnostic threshold for GDM during pregnancy on infant development involving risks of Ages and Stages Questionnaires (ASQ-3) failure including communication and personal social domains at 12 months. While Maternal GDM and fasting plasma glucose were associated with failing the communication domain in offspring, the 1-hour plasma glucose demonstrated a greater relative risk in failing the personal social domain of ASQ-3 than either the fasting plasma glucose or 2-hour plasma glucose (44).

## Discussion

Dr. Elliot Joslin, a prominent Boston diabetologist, observations in a 1921 JAMA article entitled ‘‘The Prevention of Diabetes Mellitus’’ (45) are even more relevant currently in view of the increasing global prevalence of diabetes: “it is proper at the present time to devote attention not alone to treatment, but still more … to prevention. The results may not be quite so striking or as immediate, but they are sure to come and to be important … Real headway against the ravages of a disease begins with its prevention rather than with its treatment … It is well known that diabetic patients come too late for treatment. If the disease is detected early, it is far more susceptible to diet … More energy must be exerted to discover this disease. One must hunt for it.’’

The 1-hour plasma glucose during the 75-gram OGTT has common applicability and can serve as an early biomarker for dysglycemia across the diabetes spectrum (**Table 1**). Of historical note, the National Diabetes Data Group in 1979 required three interim glucose levels during the OGTT at 30, 60, and 90 minutes (46, 47), which was modified in 1985 to the fasting and 2-hour post-load values with the 1-hour level maintained for diagnosing GDM. In retrospect, deleting the 1-plasma glucose may have been premature.

**Table 1:** Potential roles for the 1-hour plasma glucose.

1. Reduce time, labor, and costs in performing 2- hour OGTT
2. Increase convenience for patients and providers with greater utilization
3. Detect individuals at risk for progression to type 2 diabetes
4. Identify individuals at high-risk for micro- and macro-vascular complications
5. Identify individuals at high-risk for mortality
6. Diagnose individuals with type 2 diabetes
7. Identify antibody positive individuals at risk of developing type 1 diabetes for participation in prevention trials
8. Diagnose gestational diabetes
9. Predict adverse neonatal outcomes and infant development
10. Identify pregnant women at high-risk of future type 2 diabetes and CVD

Screening with a fasting plasma glucose, 2-hour plasma glucose or HbA1c are relatively insensitive for detecting dysglycemia and therefore inadequate for the early identification of high-risk individuals. Identifying high-risk individuals earlier with a more sensitive biomarker than current screening modalities, offers the opportunity for timely intervention reducing the likelihood of progression to diabetes, development of complications, and mortality. Prevention of type 2 diabetes would include intensive lifestyle modification with pharmacotherapy, principally metformin, as required. Of equal importance, early recognition of autoantibody positive individuals at high risk for type 1 diabetes based on an Index incorporating the 1-hour OGTT may benefit from recruitment into prevention trials.

Therefore, evidence from populations at risk for type 1 diabetes, type 2 diabetes or GDM substantiates the 1-hour plasma glucose as a sensitive alternative marker for identifying high-risk individuals when ß-cell function is relatively more functional. An elevated 1-hour plasma glucose with different threshold levels and contributing to a composite index in risk assessment for type 1 diabetes, could therefore diagnose dysglycemia and risk for complications across the glycemic spectrum. With agreement by scientific organizations, the 1-hour OGTT could replace the 2-hour OGTT for detecting individuals at high-risk for type 2 diabetes and furthermore may also diagnose type 2 diabetes. A 1-hour OGTT could therefore facilitate greater access to screening in clinical practice and allow for earlier diagnosis of dysglycemia.

## Data Availability Statement

The original contributions presented in the study are included in the article/supplementary material. Further inquiries can be directed to the corresponding author.

## Author Contributions

The author confirms being the sole contributor of this work and has approved it for publication.

## Conflict of Interest

The author declares that the research was conducted in the absence of any commercial or financial relationships that could be construed as a potential conflict of interest.

## Publisher’s Note

All claims expressed in this article are solely those of the authors and do not necessarily represent those of their affiliated organizations, or those of the publisher, the editors and the reviewers. Any product that may be evaluated in this article, or claim that may be made by its manufacturer, is not guaranteed or endorsed by the publisher.
